# Supplementary figures and images for: Nonlinear temperature effects on multifractal complexity of metabolic rate of mice
Source: PeerJ. 2016 Oct 20;4:e2607. doi: 10.7717/peerj.2607 (PMC5075692; doi:10.7717/peerj.2607)

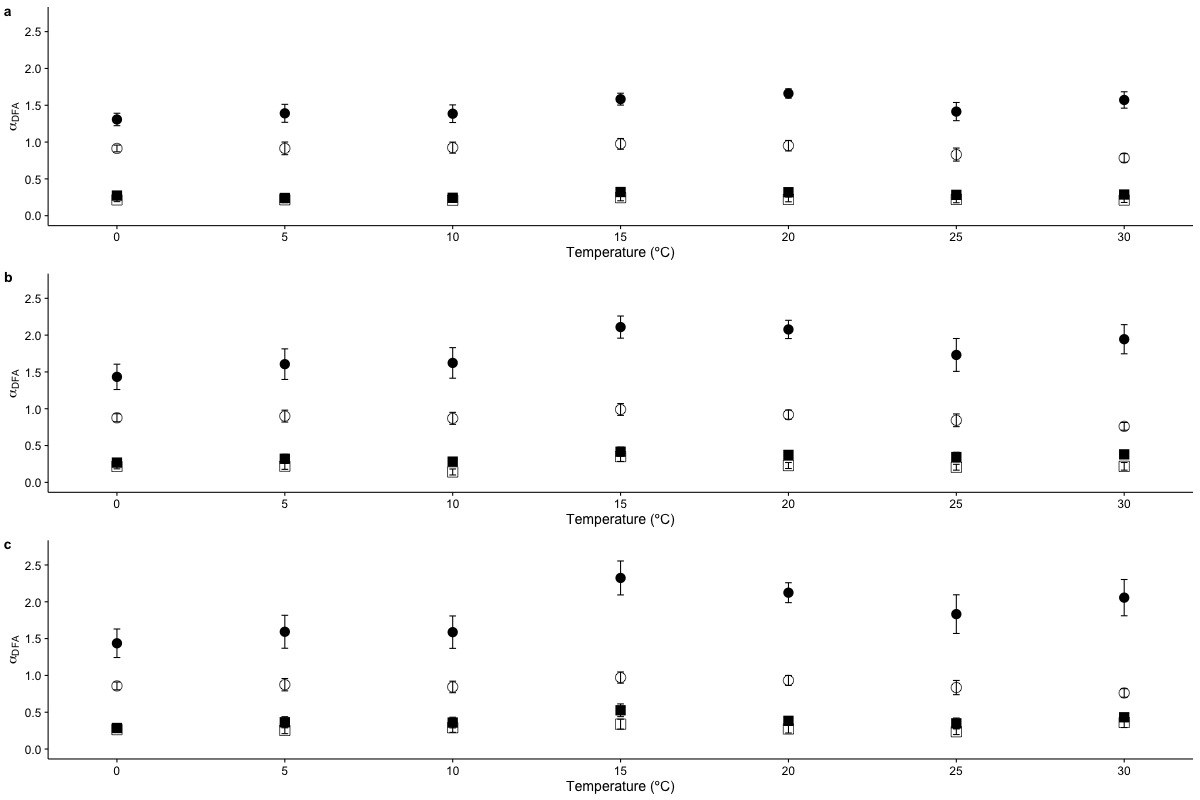

Supplement: Figure S1 — The figure shows the average DFA scaling exponent αDFA calculated as a function of experimental temperature for (a) Linear DFA de-trending, (b) quadratic polynomial de-trending and (c) cubic de-trending. Average scaling exponents corresponding to exponent for raw r(VO2) data within the 10 < s < 100 scaling regime are shown with filled circles, while filled squares show the scaling exponents for the raw r(VO2) data within the 100 < s < 1,024 scaling regimes are shown with. Open circles and squares show the scaling exponents for these two respective scaling regimes when data are shuffled. [file peerj-04-2607-s002.jpeg]

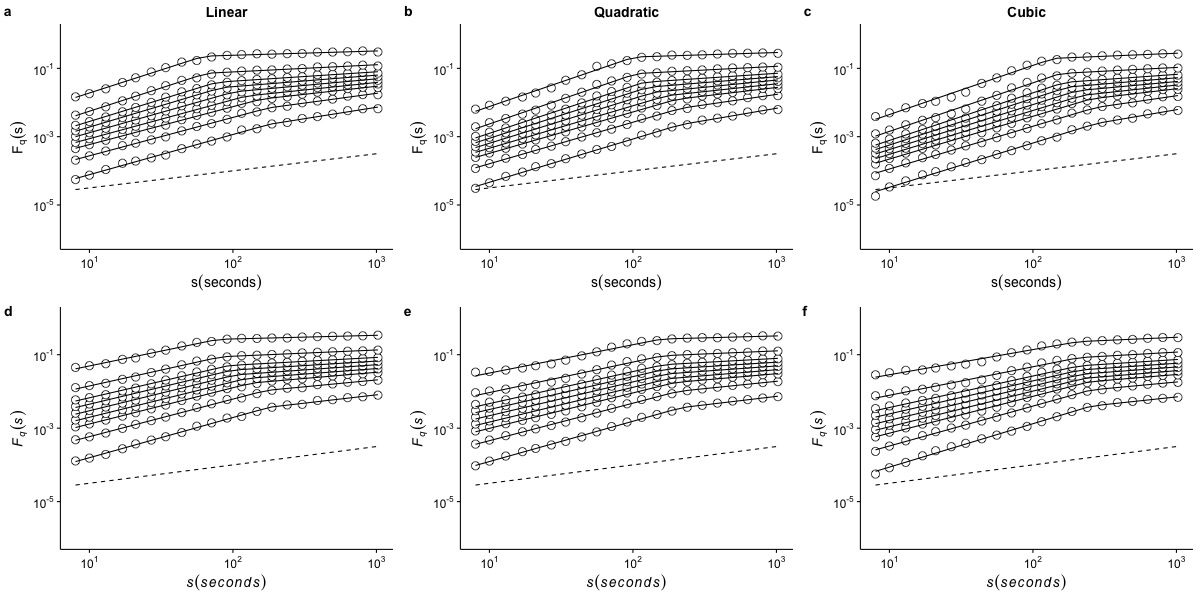

Supplement: Figure S2 — The figure shows the average generalized fluctuation function Fq(s) vs. time scale s in log–log plots for r(VO2) fluctuations using different de-trending orders. Figures in the top row show average results calculated for the raw time series measured at Ta = 0°C when data are detrended using (a) a linear function, (b) a quadratic polynomial and (c) a cubic polynomial. The bottom row shows the results for the shuffled time series when the data are detrended using (d) a linear function, (e) a quadratic polynomial and (f) a cubic polynomial. Open circles show the observed Fq(s) values for different values of q, with q = 8, 4, 2, 1, 0, −1,−2, −4, and −8 (from the top to the bottom). All curves are shifted vertically for clarity. The straight lines are best piecewise linear regression fits to the Fq(s) functions. Dashed straight lines with slope h = 0.5 are shown below the data in each figure to allow qualitative comparison with the uncorrelated case. [file peerj-04-2607-s003.jpeg]

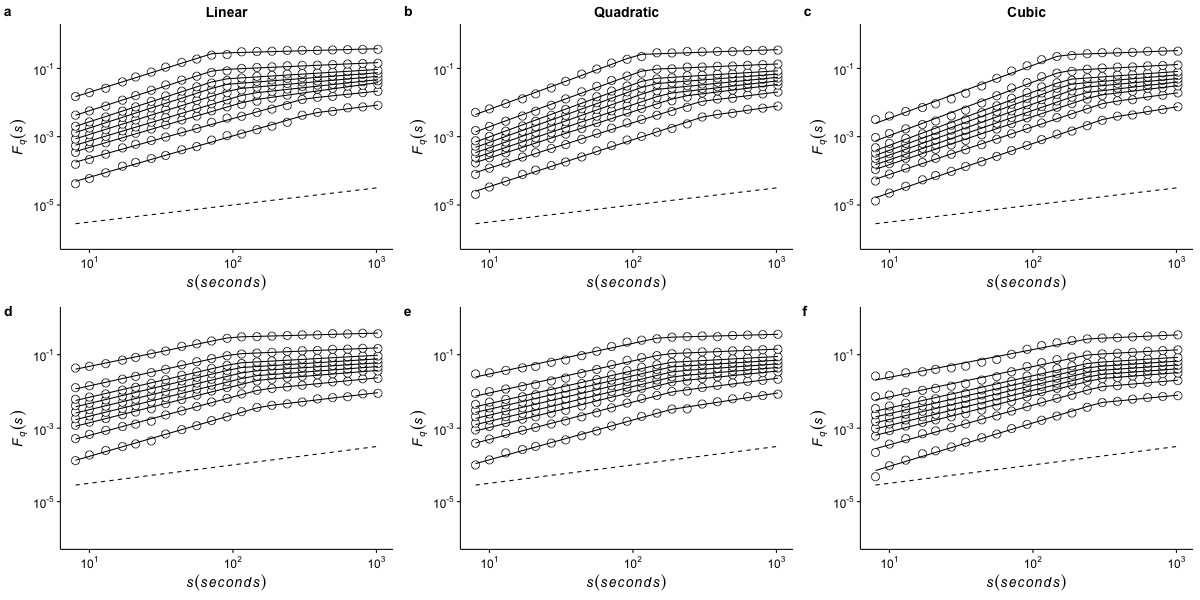

Supplement: Figure S3 — The figure shows the average generalized fluctuation function Fq(s) vs. time scale s in log–log plots for r(VO2) fluctuations using different de-trending orders. Figures in the top row show average results calculated for the raw time series measured at Ta = 5°C when data are detrended using (a) a linear function, (b) a quadratic polynomial and (c) a cubic polynomial. The bottom row shows the results for the shuffled time series when the data are detrended using (d) a linear function, (e) a quadratic polynomial and (f) a cubic polynomial. Open circles show the observed Fq(s) values for different values of q, with q = 8, 4, 2, 1, 0, −1,−2, −4, and −8 (from the top to the bottom). All curves are shifted vertically for clarity. The straight lines are best piecewise linear regression fits to the Fq(s) functions. Dashed straight lines with slope h = 0.5 are shown below the data in each figure to allow qualitative comparison with the uncorrelated case. [file peerj-04-2607-s004.jpeg]

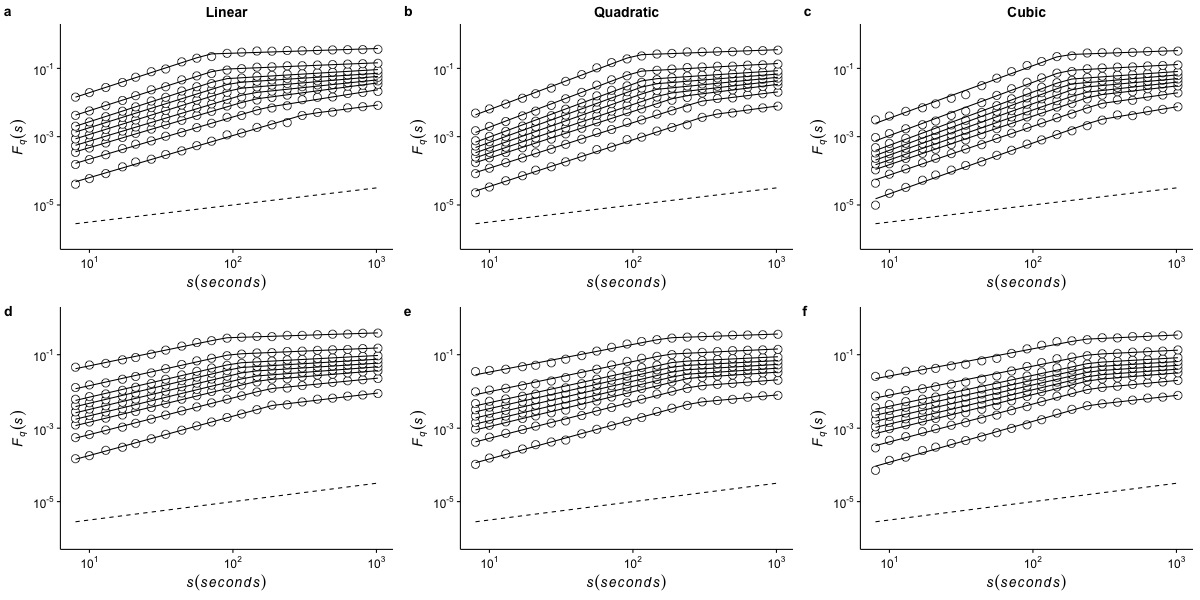

Supplement: Figure S4 — The figure shows the average generalized fluctuation function Fq(s) vs. time scale s in log–log plots for r(VO2) fluctuations using different de-trending orders. Figures in the top row show average results calculated for the raw time series measured at Ta = 10°C when data are detrended using (a) a linear function, (b) a quadratic polynomial and (c) a cubic polynomial. The bottom row shows the results for the shuffled time series when the data are detrended using (d) a linear function, (e) a quadratic polynomial and (f) a cubic polynomial. Open circles show the observed Fq(s) values for different values of q, with q = 8, 4, 2, 1, 0, −1,−2, −4, and −8 (from the top to the bottom). All curves are shifted vertically for clarity. The straight lines are best piecewise linear regression fits to the Fq(s) functions. Dashed straight lines with slope h = 0.5 are shown below the data in each figure to allow qualitative comparison with the uncorrelated case. [file peerj-04-2607-s005.jpeg]

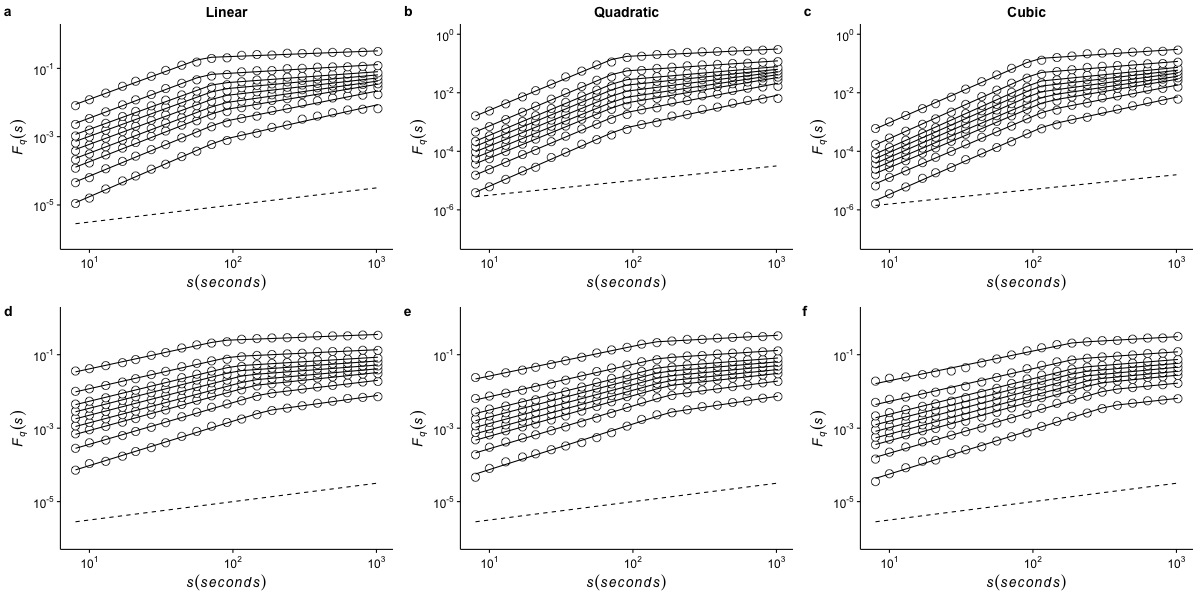

Supplement: Figure S5 — The figure shows the average generalized fluctuation function Fq(s) vs. time scale s in log–log plots for r(VO2) fluctuations using different de-trending orders. Figures in the top row show average results calculated for the raw time series measured at Ta = 15°C when data are detrended using (a) a linear function, (b) a quadratic polynomial and (c) a cubic polynomial. The bottom row shows the results for the shuffled time series when the data are detrended using (d) a linear function, (e) a quadratic polynomial and (f) a cubic polynomial. Open circles show the observed Fq(s) values for different values of q, with q = 8, 4, 2, 1, 0, −1,−2, −4, and −8 (from the top to the bottom). All curves are shifted vertically for clarity. The straight lines are best piecewise linear regression fits to the Fq(s) functions. Dashed straight lines with slope h = 0.5 are shown below the data in each figure to allow qualitative comparison with the uncorrelated case. [file peerj-04-2607-s006.jpeg]

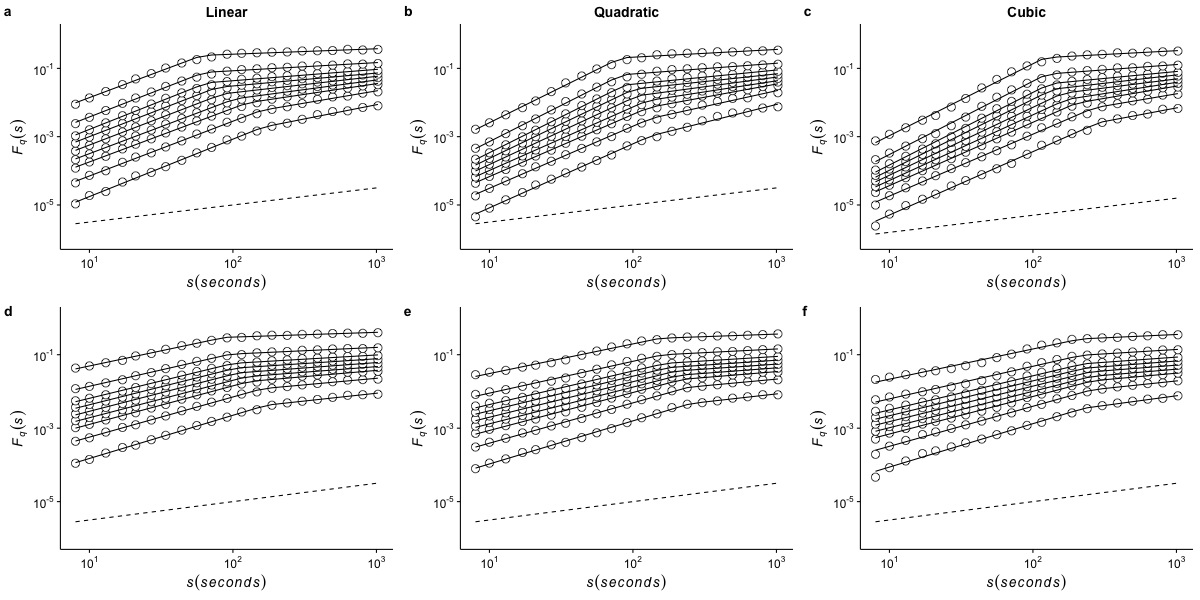

Supplement: Figure S6 — The figure shows the average generalized fluctuation function Fq(s) vs. time scale s in log–log plots for r(VO2) fluctuations using different de-trending orders. Figures in the top row show average results calculated for the raw time series measured at Ta = 20°C when data are detrended using (a) a linear function, (b) a quadratic polynomial and (c) a cubic polynomial. The bottom row shows the results for the shuffled time series when the data are detrended using (d) a linear function, (e) a quadratic polynomial and (f) a cubic polynomial. Open circles show the observed Fq(s) values for different values of q, with q = 8, 4, 2, 1, 0, −1,−2, −4, and −8 (from the top to the bottom). All curves are shifted vertically for clarity. The straight lines are best piecewise linear regression fits to the Fq(s) functions. Dashed straight lines with slope h = 0.5 are shown below the data in each figure to allow qualitative comparison with the uncorrelated case. [file peerj-04-2607-s007.jpeg]

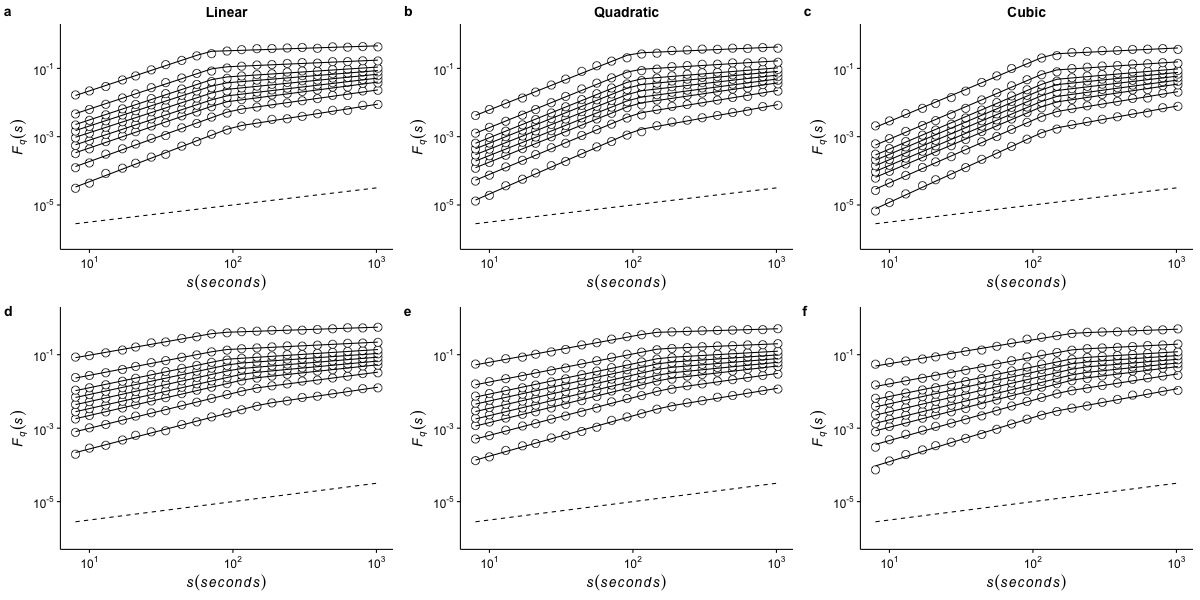

Supplement: Figure S7 — The figure shows the average generalized fluctuation function Fq(s) vs. time scale s in log–log plots for r(VO2) fluctuations using different de-trending orders. Figures in the top row show average results calculated for the raw time series measured at Ta = 25°C when data are detrended using (a) a linear function, (b) a quadratic polynomial and (c) a cubic polynomial. The bottom row shows the results for the shuffled time series when the data are detrended using (d) a linear function, (e) a quadratic polynomial and (f) a cubic polynomial. Open circles show the observed Fq(s) values for different values of q, with q = 8, 4, 2, 1, 0, −1,−2, −4, and −8 (from the top to the bottom). All curves are shifted vertically for clarity. The straight lines are best piecewise linear regression fits to the Fq(s) functions. Dashed straight lines with slope h = 0.5 are shown below the data in each figure to allow qualitative comparison with the uncorrelated case. [file peerj-04-2607-s008.jpeg]

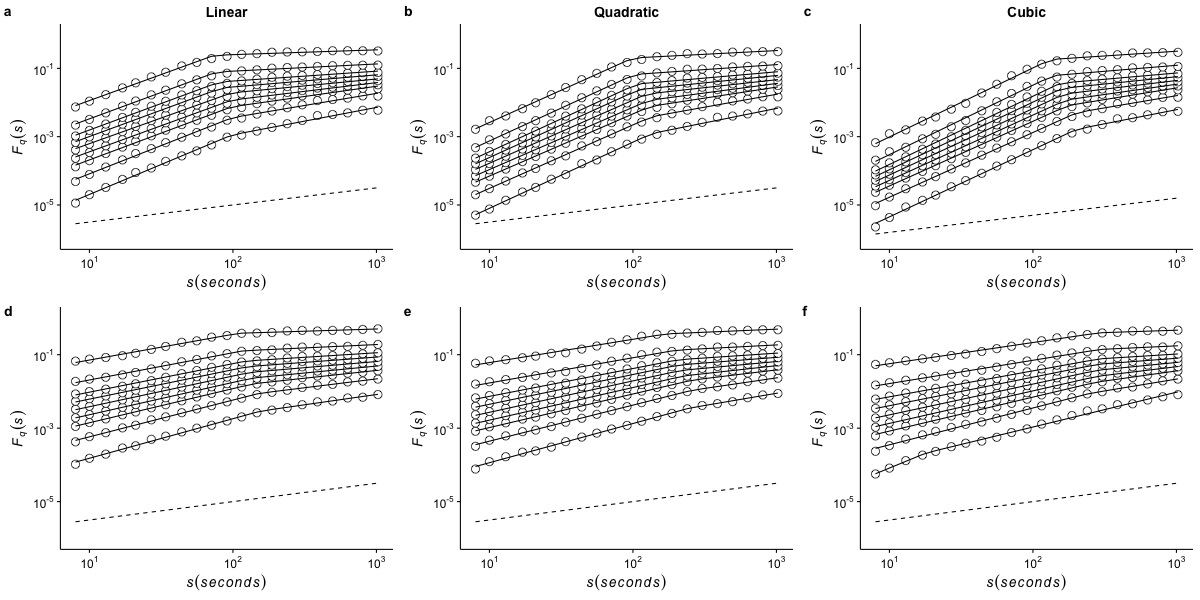

Supplement: Figure S8 — The figure shows the average generalized fluctuation function Fq(s) vs. time scale s in log–log plots for r(VO2) fluctuations using different detrending orders. Figures in the top row show average results calculated for the raw time series measured at Ta = 30°C when data are detrended using (a) a linear function, (b) a quadratic polynomial and (c) a cubic polynomial. The bottom row shows the results for the shuffled time series when the data are detrended using (d) a linear function, (e) a quadratic polynomial and (f) a cubic polynomial. Open circles show the observed Fq(s) values for different values of q, with q = 8, 4, 2, 1, 0, −1,−2, −4, and −8 (from the top to the bottom). All curves are shifted vertically for clarity. The straight lines are best piecewise linear regression fits to the Fq(s) functions. Dashed straight lines with slope h = 0.5 are shown below the data in each figure to allow qualitative comparison with the uncorrelated case. [file peerj-04-2607-s009.jpeg]

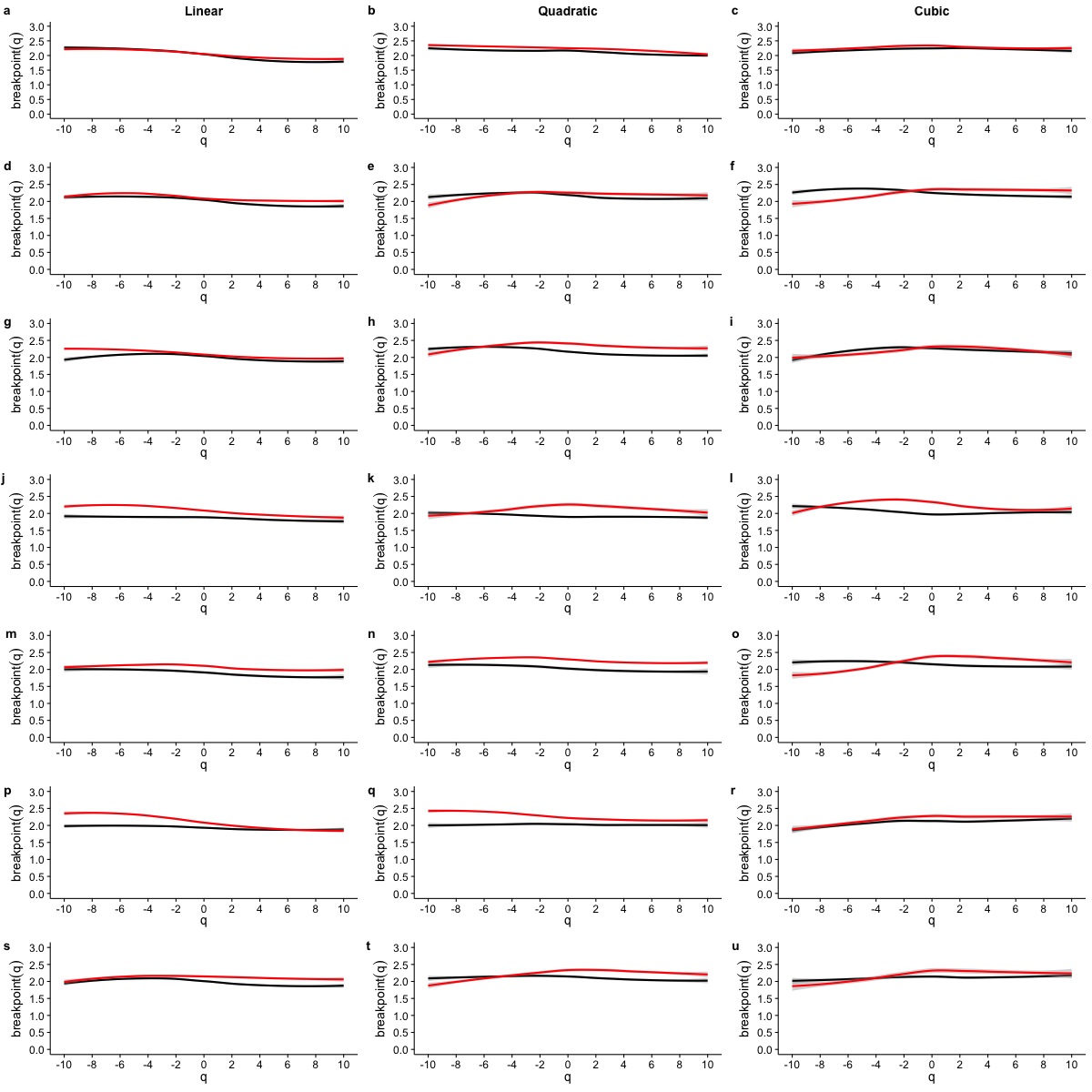

Supplement: Figure S9 — Top to bottom rows show the results for Ta = 0°C to Ta = 30°C respectively. Left hand, central and right hand columns show the results for linear, quadratic and cubic de-trending polynomials respectively. In all figures, black lines show the smoothed conditional mean estimate of the breakpoint, while red lines show the smoothed conditional mean estimates for shuffled data. [file peerj-04-2607-s010.jpeg]

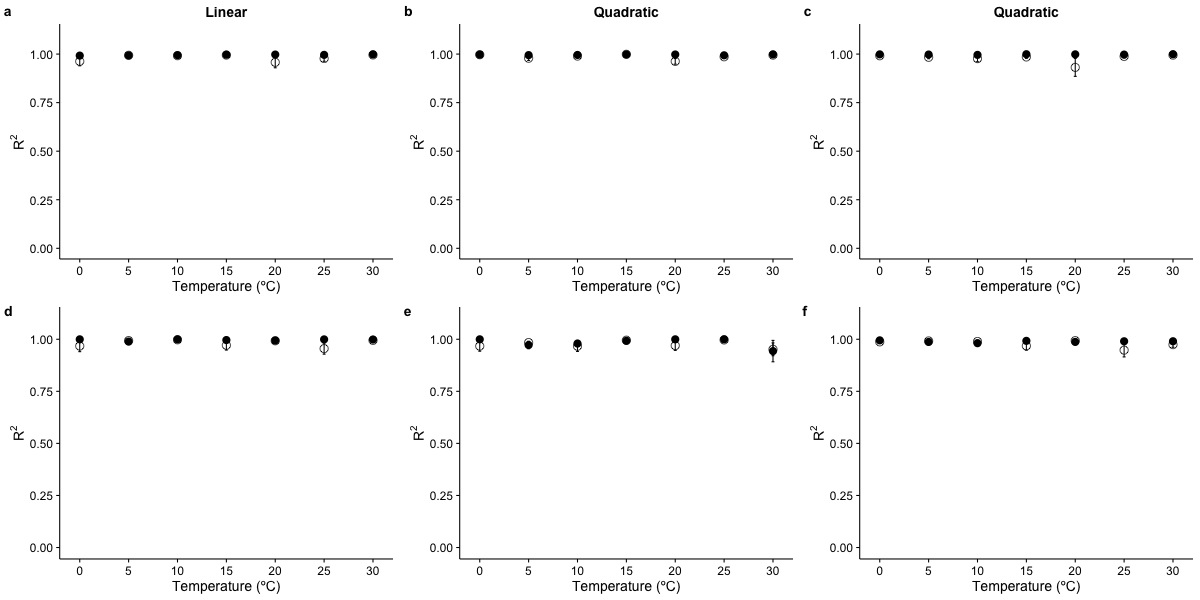

Supplement: Figure S10 — The figure shows the average coeficient of determination (R2) for the fit of Renyi exponent spectra (τ(q)) to MMCM under different temperature treatments. Average R2 value in raw r(VO2) data is shown for (a) Linear de-trending, (b) quadratic polynomial de-trending and (c) cubic de-trending. Average R2 value in AAFT shuffled r(VO2) data is shown for (d) Linear de-trending, (e) quadratic polynomial de-trending and (f) cubic de-trending. Average R2 values for r(VO2) data within the 10 < s < 100 scaling regime are shown with filled circles, while open circles show average R2 values for r(VO2) data within the 100 < s < 1,024 scaling regime. [file peerj-04-2607-s011.jpeg]

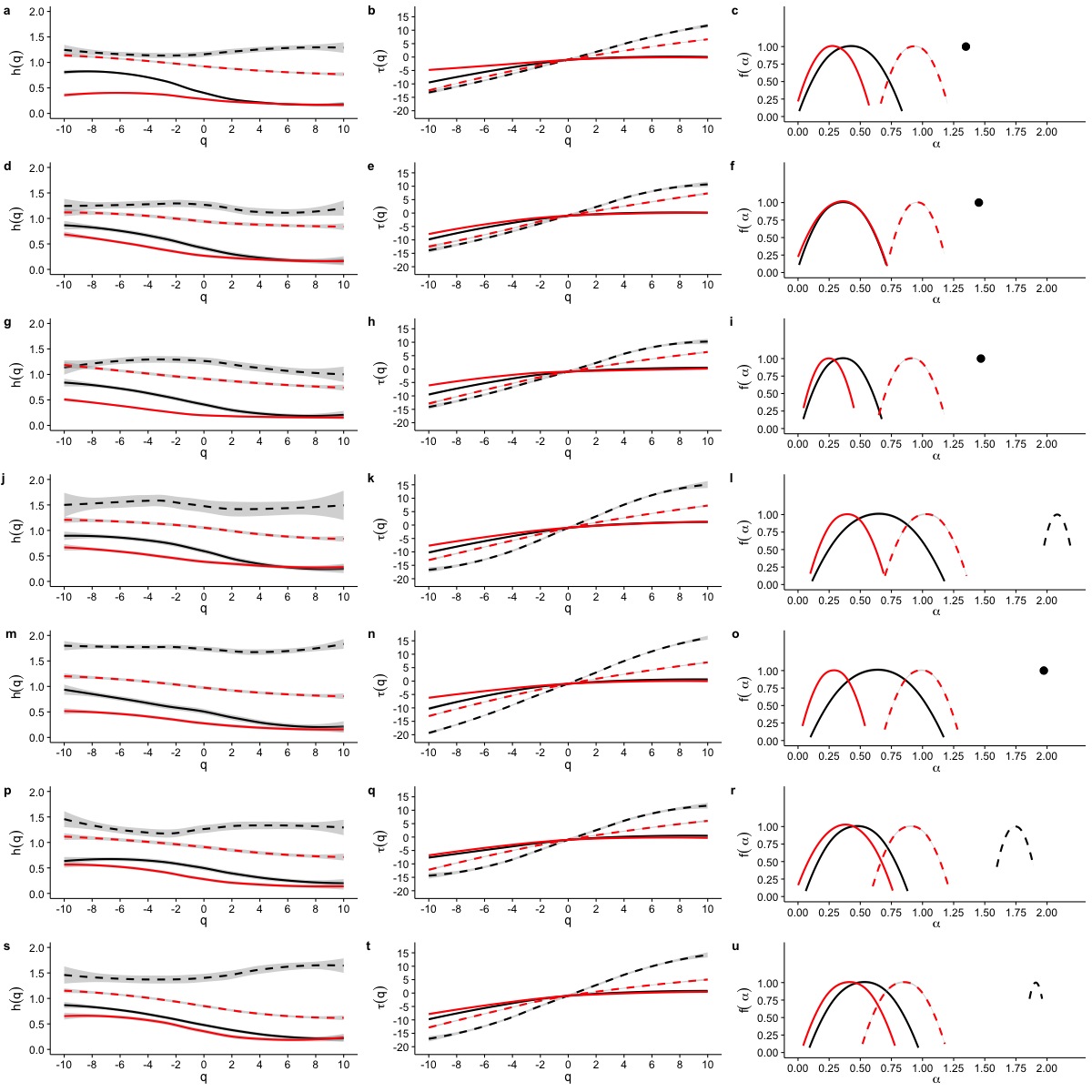

Supplement: Figure S11 — The figure shows the effects of quadratic de-trending on the multifractal scaling analysis for all mice studied. Left, central and right hand column show the results for the generalized Hurst exponent spectra (h(q)), Renyi exponent spectra (τ(q)) and singularity spectra (f(α)). Each figure shows the smoothed conditional mean of the different spectra in dashed and continuous black lines, representing data for the first and second scaling regimes respectively. For shuffled data, dashed and continuous red lines show the smoothed conditional mean of the different spectra for the first and second scaling regimes respectively. For figures (c), (f), (i) and (o), the singularity spectra of the first regime corresponds to a single point, shown by a filled circle. The singularity spectra reveal that for temperatures in the range 0°C < Ta < 10°C the time scales in the 8 < s < 100 range present a monofractal scaling, while all remaining temperatures show a weak multifractal scaling. All data for the second scaling regime show strong multifractality. [file peerj-04-2607-s012.jpeg]

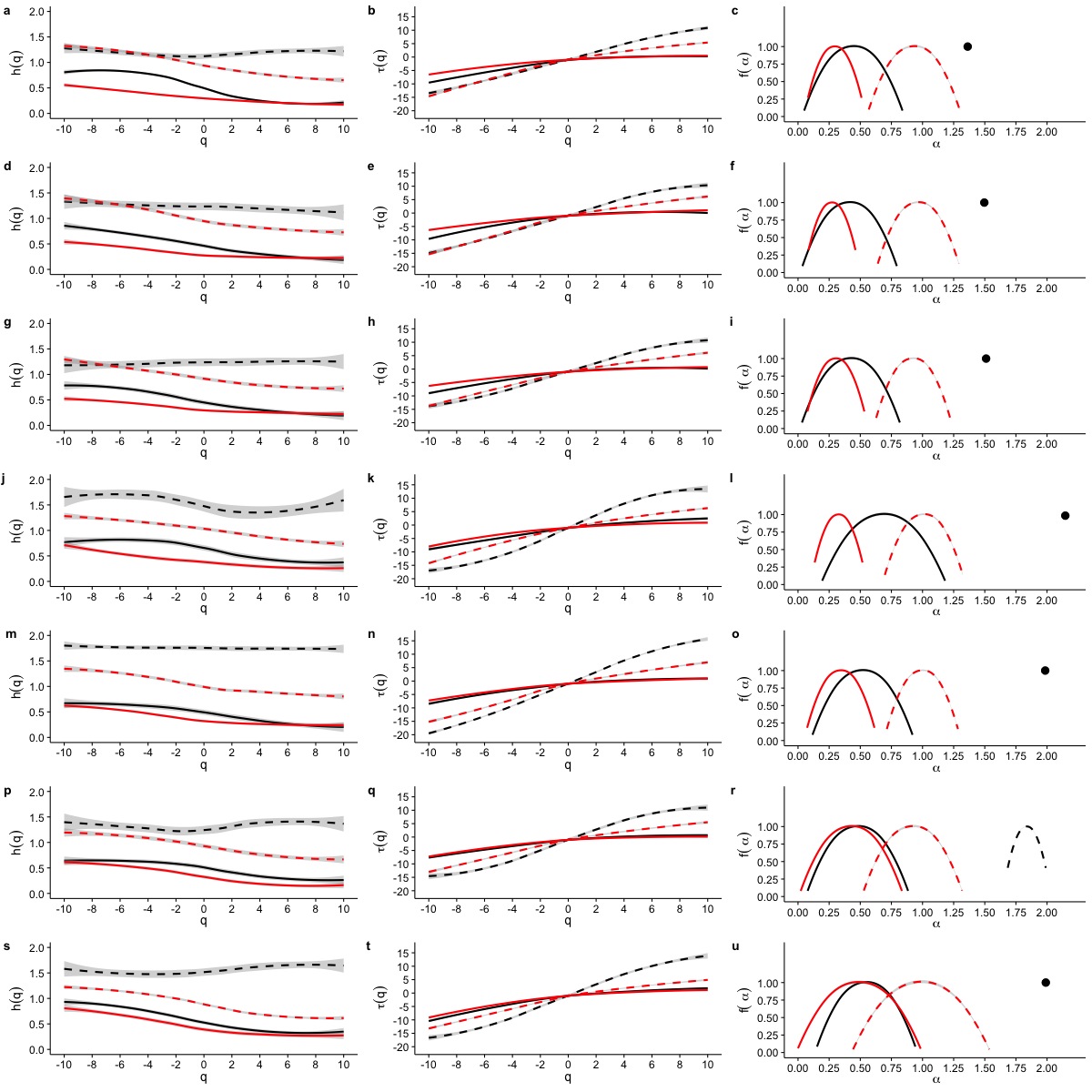

Supplement: Figure S12 — The figure shows the effects of cubic de-trending on the multifractal scaling analysis for all mice studied. Left, central and right hand column show the results for the generalized Hurst exponent spectra (h(q)), Renyi exponent spectra (τ(q)) and singularity spectra (f(α)). Each figure shows the smoothed conditional mean of the different spectra in dashed and continuous black lines, representing data for the first and second scaling regimes respectively. For shuffled data, dashed and continuous red lines show the smoothed conditional mean of the different spectra for the first and second scaling regimes respectively. For figures (c), (f), (i), (l), (o) and (u) the singularity spectra of the first regime corresponds to a single point, shown by a filled circle. The singularity spectra reveal that for all temperatures the time scales in the 8 < s < 100 range present either a monofractal scaling or a weak multifractal scaling. On the other hand, all data for the second scaling regime show strong multifractality. [file peerj-04-2607-s013.jpeg]
